# Simultaneous multi‐slice cardiac real‐time MRI at 0.55T

**DOI:** 10.1002/mrm.30364

**Published:** 2024-11-06

**Authors:** Ecrin Yagiz, Parveen Garg, Steven Y. Cen, Krishna S. Nayak, Ye Tian

**Affiliations:** ^1^ Ming Hsieh Department of Electrical and Computer Engineering, Viterbi School of Engineering University of Southern California Los Angeles California USA; ^2^ Division of Cardiology, Department of Medicine, Keck School of Medicine University of Southern California Los Angeles California USA; ^3^ Department of Neurology, Keck School of Medicine University of Southern California Los Angeles California USA; ^4^ Department of Radiology, Keck School of Medicine University of Southern California Los Angeles California USA

**Keywords:** 0.55 tesla MRI, cardiac function, real‐time MRI, simultaneous multi‐slice

## Abstract

**Purpose:**

To determine the feasibility of simultaneous multi‐slice (SMS) real‐time MRI (RT‐MRI) at 0.55T for the evaluation of cardiac function.

**Methods:**

Cardiac CINE MRI is routinely used to evaluate left‐ventricular (LV) function. The standard is sequential multi‐slice balanced SSFP (bSSFP) over a stack of short‐axis slices using electrocardiogram (ECG) gating and breath‐holds. SMS has been used in CINE imaging to reduce the number of breath‐holds by a factor of 2–4 at 1.5T, 3T, and recently at 0.55T. This work aims to determine if SMS is similarly effective in the RT‐MRI evaluation of cardiac function. We used an SMS bSSFP pulse sequence with golden‐angle spirals at 0.55T with an SMS factor of three. We cover the LV with three acquisitions for SMS, and nine for single‐band (SB). Imaging was performed on 9 healthy volunteers and 1 patient with myocardial fibrosis and sternal wires. A spatio‐temporal constrained reconstruction is used, with regularization parameters selected by a board‐certified cardiologist. Images were quantitatively analyzed with a normalized contrast and an Edge Sharpness (ES) score.

**Results:**

There was a statistically significant 2‐fold difference in contrast between SMS and SB and no significant difference in ES score. The contrast for SMS and SB were 13.38/29.05 at mid‐diastole and 10.79/22.26 at end‐systole; the ES scores for SMS and SB were 1.77/1.83 at mid‐diastole and 1.50/1.72 at end‐systole.

**Conclusions:**

SMS cardiac RT‐MRI at 0.55T is feasible and provides sufficient blood‐myocardium contrast to evaluate LV function in three slices simultaneously without any gating or periodic motion assumptions.

## 1 INTRODUCTION

Real‐time MRI (RT‐MRI) involves the continuous acquisition of images without any reliance on gating, synchronization, or repetition of the underlying movement.^1^ This makes it possible to visualize dynamic processes with minimal assumptions. RT‐MRI is an appealing strategy for MRI‐guided interventions^2^ and diagnostic applications that involve irregular movement, such as musculoskeletal, upper airway, gastrointestinal, pediatric, and cardiac imaging.^3^, ^4^, ^5^, ^6^, ^7^, ^8^ Cardiac RT‐MRI has proven especially valuable during physiological stress testing (e.g., MRI‐compatible bicycle ergometer),^9^, ^10^, ^11^ in patients with arrhythmia where cardiac gating fails,^4^, ^10^ and in patients who cannot comply with breath‐hold instructions, such as children or the incapacitated.

Cardiac CINE MRI is routinely used to evaluate left ventricular function, wall motion, and regional wall thickening. The preferred pulse sequence is balanced SSFP (bSSFP), as it provides excellent SNR efficiency and blood–myocardium contrast. Standardized protocols cover the whole heart over multiple breath‐holds with a stack of short axis slices (10–12 slices).^12^ Simultaneous multi‐slice (SMS) techniques have been applied to accelerate cardiac applications. While a majority of the applications employ spoiled gradient‐echo sequences,^13^, ^14^, ^15^, ^16^, ^17^ in cardiac CINE, SMS bSSFP techniques have been used to reduce the number of breath‐holds by a factor of 2–4 at 1.5T, 3T, and most recently at 0.55T.^18^, ^19^, ^20^, ^21^

Cardiac RT‐MRI is typically performed for 2D slices,^22^, ^23^ with the option to time‐interleave multiple slices^24^, ^25^ at the cost of reduced temporal resolution. In this work, we employ an SMS factor of 3 and cover the left ventricle (LV) in three acquisitions, that is, nine slices. We employ a spiral bSSFP acquisition with parameters that leverage the strengths of the mid‐field system (0.55T).^21^ The three simultaneously acquired slices, if chosen in accordance with standard views, can provide simultaneous coverage of 16 (out of 17) left ventricular myocardial segments, according to the American Heart Association Standardized Myocardial Segmentation and Nomenclature for Tomographic Imaging of the Heart.^26^

In this work, we demonstrate the feasibility of SMS cardiac RT‐MRI at 0.55T. We demonstrate that cardiac functional assessment can be achieved in one‐third of the scan time (compared to conventional single‐band imaging) and that this technique can capture irregular rhythms simultaneously for apical, mid, and basal short‐axis slices, with a temporal resolution of 45 ms.

## 2 METHODS

### 2.1 Experimental methods

Experiments were performed on a whole‐body 0.55T system (prototype MAGNETOM Aera, Siemens Healthineers, Erlangen, Germany) with high‐performance shielded gradients (45 mT/m amplitude, 200 T/m/s slew rate).^27^ Imaging was performed using the RTHawk system (Vista.AI Inc., Menlo Park, CA).^28^ The body coil was used for RF transmission, and a six‐channel surface body coil (anterior) and six elements from an 18‐channel spine array (posterior) were used for signal reception. Data were collected from nine healthy volunteers (three females/six males, aged 28.4 ± 8.6 y, body mass index (BMI) 23.7 ± 3, heart rate 73.1 ± 13 bpm) and one patient (male, 40 y, BMI 34.0, heart rate 70 bpm) with myocardial fibrosis and sternal wire sutures. All subjects were scanned after providing written informed consent under protocols approved by our Institutional Review Board.

### 2.2 Acquisition

We used a blipped CAIPI SMS bSSFP pulse sequence with golden‐angle spiral readouts based on Tian et al.^21^ A single‐slice RF excitation pulse was superimposed with frequency‐modulated versions to form an SMS RF‐pulse.^29^ A conservative peak B1 value of 19 μT was used to allow for scanning subjects with different body habitus, as the maximum achievable B1 is calculated for each scan during pre‐scan calibration. The readout was an M1‐nulled uniform‐density spiral trajectory with a duration of 2.7 ms.^30^ Scan parameters: spatial resolution = 2.2 × 2.2 mm^2^, slice thickness = 8 mm, TE/TR = 1.33/6.6 ms for SMS, and 0.72/5.3 ms for single‐band (SB). Note that the SMS excitation pulse is longer due to the peak B1 constraints, causing SMS acquisitions to have a slightly longer TE and TR. The flip angle (FA) was set to 100°, experimentally chosen for optimal blood‐myocardium contrast for bSSFP cardiac cine imaging at 0.55T.^21^

To explore the effect of spatial resolution on image quality, additional SMS scans were performed with in‐plane spatial resolutions 1.2 × 1.2 mm^2^, 1.5 × 1.5 mm^2^, and 1.8 × 1.8 mm^2^ in two volunteers (one female, age 21, BMI 20, heart rate 85; one male, age 49, BMI 29, heart rate 73). The readout durations and TE were kept constant, and the TRs were 7.22, 6.96, and 6.77 ms, respectively, due to the slightly longer rewinder needed at finer spatial resolutions.

**FIGURE 1:**
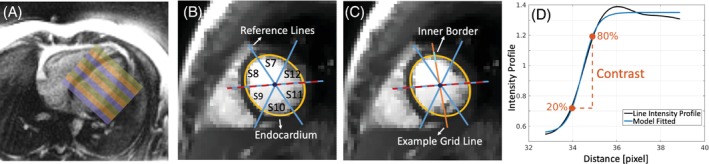
Illustration of slice positioning, detection of myocardial segments, and calculation of contrast and ES. (A) LV coverage and slice positioning. All imaging was done in the short‐axis orientation, with the entire LV covered in nine slices. The color blocks indicate the slices acquired simultaneously. (B) Semi‐automated segmentation of endocardial border (yellow) and one manually‐placed reference line (dashed). The other lines are automatically generated. (C) One of the lines used for edge sharpness (orange) covering S7 and S10. (D) Representative line intensity profile (black) with its sigmoid fit (blue). The ES is identified as the slope of the fitted sigmoid function (s); the higher, the sharper the edge. Then, normalized contrast is calculated as Contrast=I80−I20σ, where $I_{x}$ is the x%‐ of the maximum signal intensities, and σ is derived from fully‐sampled gridded images for normalization.

All imaging was performed in the short‐axis orientation, with the entire LV covered in nine parallel slices with 8 mm spacing, as shown in Figure 1A. For SB, each slice was acquired separately for 15 s per slice. For SMS, the same nine slices were collected in three sets of three simultaneous slices for 15 s per set.

### 2.3 Reconstruction

All images were reconstructed using spatiotemporal constrained reconstruction (STCR) that used gradient impulse response function (GIRF) corrected spiral trajectory.^31^, ^32^ The following cost function was minimized using a nonlinear conjugate gradient algorithm with a line search:

$${\left\|\mathrm{Am}-d\right\|}_{2}^{2}+\lambda_{s}{\left\|\sqrt{{\left(\nabla_{x}m\right)}^{2}+{\left(\nabla_{y}m\right)}^{2}+\varepsilon }\right\|}_{1}+\lambda_{t}{\left\|\sqrt{{\left(\nabla_{t}m\right)}^{2}+\varepsilon }\right\|}_{1}$$

where d is multi‐coil k‐space data, A=ΦFS is the encoding matrix with the Φ phase modulation for CAIPI (included only for SMS reconstruction), F non‐uniform Fourier transform, and S coil sensitivities estimated by Walsh method.^33^ m represents the image series to be reconstructed, $\lambda_{t,s}$ are temporal and spatial regularization parameters, respectively, and ε is a small positive value to avoid singularity issues. The regularization parameters ($\lambda_{t}=0.05C,\lambda_{s}=0.005C$ where C indicates the scaling factor, which is the highest pixel intensity in $A^{-1}d$) were chosen from a parameter sweep followed by a qualitative assessment by a board‐certified cardiologist experienced with cardiac MRI. The temporal resolution was set to 45 ms/frame for both SMS and SB images, corresponding to six and eight spiral arms per frame, respectively.

All images were reconstructed offline in MATLAB 2021a (The MathWorks, Inc. Natick, MA) on a server equipped with 4x AMD EPYC 7502 32‐core CPU and 4x NVIDIA A100 GPU (40Gb memory for each). The iterative part of the reconstruction was performed on a single GPU core, while the rest was performed on the CPU. The reconstruction for 2.2 × 2.2 mm^2^ (matrix size of 112 × 112) takes 0.56 s/frame for SMS and 0.23 s/frame for SB. For an acquisition of 15 s (˜330 frames), the reconstruction takes 200 s for SMS and 75 s for SB.

### 2.4 Evaluation

Image quality was assessed quantitatively by a normalized contrast and by an edge sharpness (ES) score in manually selected systolic and diastolic frames. First, a representative mid‐ventricular short‐axis slice was selected for each volunteer, which included papillary muscles as a visual landmark. Then, the myocardium was segmented in a semi‐automatic fashion according to the Standardized Myocardial Segmentation guide using an in‐house MATLAB script.^26^

Figure 1B shows an example of this procedure with the placement of the endocardium border, reference lines, and lines for the intensity profiles. The endocardium border was manually placed using an ellipsoid model. Then, a reference line (dashed line) was manually identified such that it approximately divides the anteroseptal and inferoseptal myocardium into two equal parts (between segments 8 and 9). Two more reference lines were automatically placed by rotating the first line by 60° and 120°, respectively, to divide the myocardium into six segments (S7–S12). Finally, $N_{l}=50$ lines for the ES calculation were automatically placed within each segment uniformly; Figure 1C shows one representative line passing over segments 7 and 10.

Once the lines were placed, the line segments across the endocardium border were extracted. Then, the line intensity profiles were obtained and interpolated to a 100x finer grid. After, a sigmoid function was fitted for each line segment. Figure 1D shows a representative line intensity profile together with its model fitting. The slope of this sigmoid (s) is assigned as the ES score; the higher the value, the sharper the edge.^34^

Then, the 20%‐ and 80%‐ of the maximum signal intensities ($I_{20}$ and $I_{80}$) were recorded on the fitted line intensity. The thermal noise standard deviation (σ) was estimated from a region of interest placed in the background (avoiding tissue signal and any residual motion artifact) of gridding‐reconstructed images using 144 arms/frame (exceeding the Nyquist criterion) for each dataset. This was followed by image intensity scaling from the LV blood to match the intensity of the iterative reconstruction. Normalized contrast is computed as Contrast=I80−I20σ. The same procedure was applied to all SMS and SB images, and the manual placements were drawn at similar positions.

We performed statistical analysis of the normalized contrast and ES scores separately by using a hierarchical Poisson regression model with generalized estimating equations.^35^ Then, we compared six myocardium segments (S7–S12) between the SMS and SB acquisitions at end‐diastole and end‐systole. We have controlled the false discovery rate by the Benjamini‐Hochberg procedure.^36^

## 3 RESULTS

Videos S1 and S2 show the resolution sweep performed on two volunteers (one female, age 21, BMI 20, heart rate 85; one male, age 49, BMI 29, heart rate 73). Video S1 demonstrates good blood‐myocardium contrast even at finer resolutions. We opted for an in‐plane resolution of 2.2x2.2 mm^2^ to accommodate a variety of body habitus.

**FIGURE 2:**
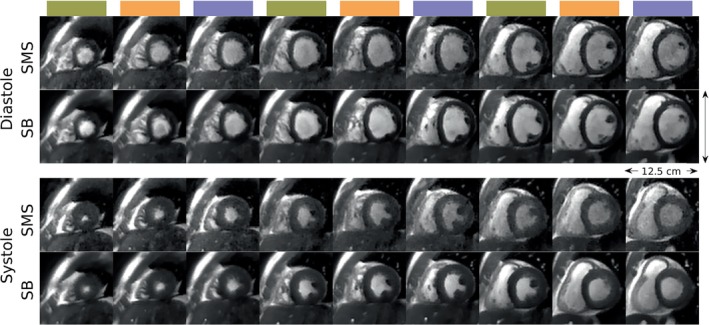
Representative SB and SMS images at mid‐diastole and end‐systole. The color blocks indicate the SMS slices acquired simultaneously, matching Figure 1A. At mid‐diastole, the SMS and SB image contrasts are comparable. At end‐systole, SMS exhibits lower blood‐myocardium contrast than SB. This reduction did not hinder the evaluation of wall motion, volumes, or ejection fraction. Video S3 shows the SB and SMS results synchronized over one heartbeat.

Figure 2 shows representative SB and SMS images from one healthy volunteer at mid‐diastole and end‐systole. The color blocks indicate the SMS slices acquired simultaneously, matching Figure 1A. The SMS images provide sufficient blood‐myocardium contrast and capture the underlying dynamics. At mid‐diastole, SMS and SB images have a comparable blood‐myocardium contrast visually. At end‐systole, SMS exhibits lower contrast than SB. Video S3 shows the corresponding videos synchronized over one heartbeat.

**FIGURE 3:**
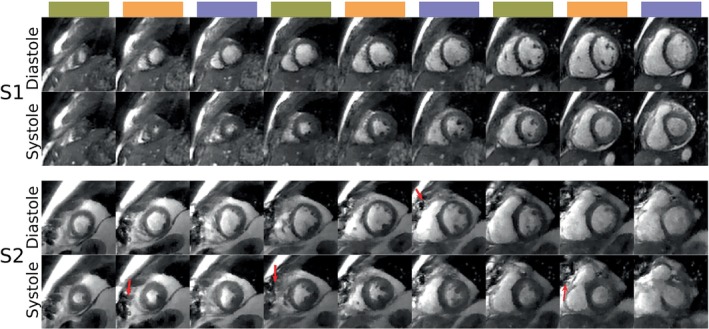
SMS images from mid‐diastole and end‐systole from two subjects with different body habitus. The top rows show a healthy volunteer (S1, female, 21 y old, BMI 20, heart rate 85 bpm). The bottom rows show a patient (S2, male, 40 y old, BMI 34, heart rate 70 bpm) with a history of myocardial infarction and sternal wire sutures (red arrows). The metal artifacts are contained in size due to lower susceptibility at mid‐field. Video S4 shows the corresponding full videos synchronized over one heartbeat, where the LV wall motion abnormalities can be seen in volunteer S2.

Figure 3 shows SMS images from two subjects with different body habitus. The top row, S1, is from a healthy volunteer (female, 21 y old, BMI 20, heart rate 85 bpm). The bottom row, S2, is from a patient (male, 40 y old, BMI 34, heart rate 70 bpm) with a history of myocardial fibrosis and sternal wire sutures, shown by red arrows. In both cases, the proposed method provided sufficient blood‐myocardium contrast to capture the underlying dynamics. For S2, the metal artifacts are contained in size due to lower susceptibility at mid‐field,^37^ causing no problem for the SMS. Video S4 shows the corresponding full videos synchronized over a heartbeat, where LV wall motion abnormalities can be seen in volunteer S2. Figure S1 shows the line intensity profiles for one SMS group and the corresponding SB acquisitions for the same volunteers.

**FIGURE 4:**
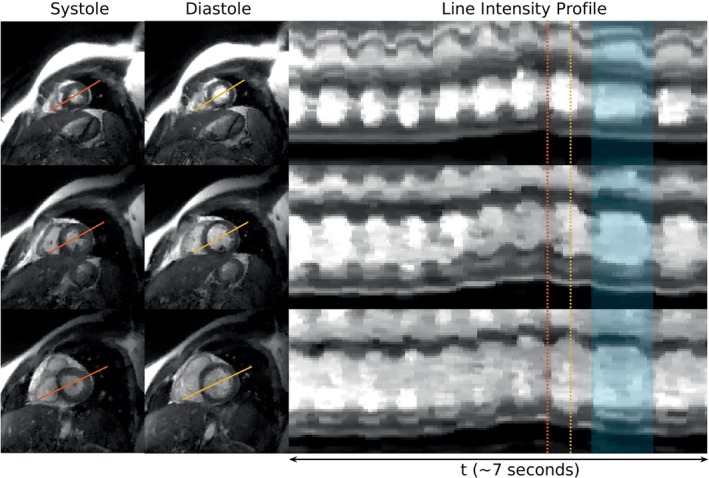
SMS images and intensity vs. time plots from a volunteer experiencing irregular rhythms. The delayed beat is highlighted in the line‐intensity plots (blue overlay), and selected systolic and diastolic frames are marked with dashed lines (red and yellow, respectively). Irregularity is captured in all three slices without manual synchronization, showing it in apical, mid, and basal short‐axis slices. Video S5 shows approximately 7 s of real‐time SMS acquisition.

Figure 4 shows SMS images and intensity vs. time plots from a volunteer experiencing irregular rhythms during the scan. The delayed beat is highlighted in the line‐intensity plots. Irregularity was captured in all three slices simultaneously without manual synchronization, showing premature ventricular contractions (PVCs) in apical, mid, and basal short‐axis slices. Video S5 shows the full video of real‐time SMS images for approximately 7 s.

Table 1 shows the normalized contrast and ES scores for each cardiac segment at mid‐diastole and end‐systole for SMS and SB acquisitions averaged across all volunteers. The average normalized contrast was 13.38/29.05 at mid‐diastole and 10.79/22.26 at end‐systole for SMS and SB, respectively. The average ES was 1.71/1.83 at mid‐diastole and 1.50/1.72 at end‐systole. The lowest contrast and ES scores, as expected, are positioned in the segments next to the mid‐cavity and on the inferior side (Segments 10–12), where the papillary muscles are situated. The highest scores are positioned in the anterior, anteroseptal, and inferoseptal (Segments 7–9). We found approximately a two‐fold difference in the contrast between the SB and the SMS images. The lowest mean contrast (averaged across the myocardium) is 10.79 (end‐systole SMS). The lowest individual scores are in Segment 12 (end‐systole SMS). The contrast of SMS and SB acquisitions was found to be statistically significantly different in all segments after Benjamini‐Hochberg correction for multiple comparisons. The difference between the ES score of SMS and SB acquisitions was not statistically significant (*p* < 0.05) after corrections, indicating a preserved edge.

**TABLE 1:** Normalized contrast and **ES** score results calculated at a mid‐ventricular short‐axis slice for each cardiac segment at manually selected systolic and diastolic frames.

	Normalized contrast				ES			
	SMS Diastole	SB Diastole	SMS Systole	SB Systole	SMS Diastole	SB Diastole	SMS Systole	SB Systole
S7	12.32 ± 6.79	27.27 ± 16.59	10.67 ± 6.17	23.42 ± 13.40	1.50 ± 1.01	1.68 ± 1.07	1.42 ± 0.87	1.37 ± 0.90
S8	16.03 ± 8.06	30.51 ± 18.13	13.65 ± 5.50	23.56 ± 13.35	2.86 ± 0.69	2.90 ± 0.74	1.69 ± 0.78	2.28 ± 0.92
S9	15.61 ± 7.72	32.46 ± 17.20	14.16 ± 5.98	31.12 ± 12.85	2.29 ± 0.72	2.55 ± 0.67	1.50 ± 0.65	1.88 ± 0.73
S10	12.38 ± 6.79	28.57 ± 16.59	9.54 ± 6.78	19.20 ± 12.80	1.41 ± 1.05	1.33 ± 0.89	1.23 ± 1.25	1.57 ± 1.32
S11	12.20 ± 8.06	27.75± 18.13	9.75 ± 3.55	21.00 ± 8.00	1.04 ± 0.80	0.85 ± 0.59	1.16 ± 1.08	1.13 ± 1.00
S12	11.70 ± 7.72	27.76 ± 17.20	6.97± 6.01	15.29 ± 12.75	1.50 ± 1.52	1.63 ± 1.35	2.00 ± 2.53	2.11 ± 1.90
AVG	**13.38** ± **7.46**	**29.05** ± **17.19**	**10.79** ± **6.26**	**22.26** ± **13.25**	**1.77** ± **1.18**	**1.83** ± **1.16**	**1.50** ± **1.38**	**1.72** ± **1.26**

*Note*: The procedure was performed on all SB and SMS images. The scores were averaged across all volunteers and shown with respective sample SDs.

Figure S2 shows SMS images and ES scores from the two volunteers in Figure 3. S1 has an average contrast of 17.59 at mid‐diastole and 12.15 at end‐systole, which was typical among subjects in this study. S2 has the lowest contrast in this study, with 7.79 at mid‐diastole and 7.65 at end‐systole. This represents the worst‐case performance.

## 4 DISCUSSION

SMS RT‐MRI images exhibited greater visual temporal variability in LV blood pool signal compared to SB RT‐MRI. We attribute this to spin history effects, specifically partial saturation of spins flowing perpendicular to the slice that has been excited by other bands in the multi‐band excitation during previous TRs. This variability did not adversely impact the visualization of the endocardial border in our study; however, we have experimentally verified (not shown) that this can be mitigated by using lower SMS flip angles, which also sacrifice blood‐myocardium contrast‐to‐noise ratio (CNR).

SMS real‐time images exhibit similar ES scores but lower contrast compared to SB. A reduced blood‐myocardium contrast in SMS compared to SB was previously reported by Tian et al.,^21^ mainly due to the SMS pulse saturating a portion of the in‐flowing blood signal. In two volunteers that are with the highest and lowest contrast, we performed an automatic segmentation (Medviso, Segment for Research) of the epicardial and endocardial borders.^38^ Video S6 shows the segmentation results over a heartbeat for all SMS and SB slices. A closely matched quantitative measurement of end‐systolic volume and end‐diastolic volume between SMS and SB suggests the SMS images provide sufficient quality for automatic segmentation. We did not perform a systematic quantitative analysis of functional parameters as this study was performed in healthy volunteers to demonstrate feasibility. A follow‐up study involving a diverse patient cohort and evaluation of functional parameters such as ejection fraction and LV volumes is needed to determine if SMS RT‐MRI can be a suitable replacement for SB RT‐MRI.

We have observed that SMS‐RT can be acquired with finer in‐plane resolutions than 2.2 × 2.2 mm^2^. However, image quality is dependent on the subject body habitus, with SNR being lower when cardiac structures are further from receiver coils, such as in obese subjects. To accommodate a diverse range of subjects, we opted for a coarser resolution in this study.

In a few isolated cases, we noticed “ripple‐like” artifacts in the SMS real‐time images. One example is Video S7. This can be attributed to residual cross‐talk between the slices, and/or a region of high signal intensity (such as blood or fat) that is outside the primary FOV and produces an aliasing artifact. The latter artifact can be reduced by employing a spiral aliasing reduction method^39^ that requires manual or semi‐automated identification of the signal hot‐spot and additional reconstruction time. We have tested two approaches to identify aliasing sources. The first approach involves manual masking over the heart area in all three slices, and we treated everything else as aliasing sources. The second approach treats the other two SMS slices as aliasing sources, requiring a tripled reconstruction time. Preliminary results shown in Video S7 suggest both approaches can effectively reduce the aliasing artifacts. Further evaluation of different reconstruction algorithms by numerical simulation, as done in^16^, ^40^ is of interest. These will include reconstruction approaches that could potentially improve slice separation and image quality, such as including slice separation regularizations^16^, ^40^ or deep learning‐based dealiasing.^41^, ^42^, ^43^, ^44^, ^45^ It is also noted from Videos S1 and S2 that the aliasing and noise level increase at the basal slices. As the imaging plane goes toward the basal direction, blood flow increases, and the signal generation area is larger. These could lead to more noise and aliasing artifacts.^39^

One limitation of our contrast analysis is that the noise estimation was based on the gridded images, which primarily reflects the acquisition noise. Analyzing the impact of regularized reconstruction algorithms on the local noise distribution is an active research area and beyond the scope of this manuscript. Another limitation of our ES and contrast analysis is the use of a single mid‐ventricular slice. The papillary muscles help with the semi‐automatic segmentation. However, they also contribute to the reduced ES score on the cavity side (Segments 10–12), as shown in Figure S2. As seen in volunteer S2 at end‐systole, the ES score is lower not only due to the reduced blood‐myocardium contrast but also, in some cases, the method measures papillary muscle‐myocardium contrast instead of blood‐myocardium contrast. This contributes to the underestimation of the true edge sharpness and the contrast.

## 5 CONCLUSIONS

We have demonstrated that SMS cardiac RT‐MRI is feasible at 0.55T and can shorten the cardiac function evaluation by a factor of three. It may provide the speed, resolution, and contrast to simultaneously evaluate left ventricular function qualitatively in three slices. It may improve the evaluation of cardiac function and regional wall motion in patients with arrhythmia (not demonstrated). This new capability is partially enabled by using time‐efficient long‐readout spirals with SNR‐efficient bSSFP imaging (due to the relaxed off‐resonance at mid‐field) and high flip‐angle multi‐band excitations (due to the relaxed SAR constraints at mid‐field). This combination of features makes the approach uniquely well‐suited for the new generation of high‐performance mid‐field MRI systems.

## Supporting information


**Video S1:** In‐plane resolution sweep performed on a healthy volunteer (female, age 21, BMI 20, heart rate 85). Each acquisition was synchronized over one heartbeat. The color‐coded blocks represent the SMS slices acquired simultaneously, matching Figure 1A. The videos demonstrate good image quality and blood‐myocardium contrast even with finer resolutions (slightly worse in basal slices as the in‐plane resolution gets finer).

**Video S2:** In‐plane resolution sweep performed on a healthy volunteer (male, age 49, BMI 29, heart rate 73). Each acquisition was synchronized over one heartbeat. The color‐coded blocks represent the SMS slices acquired simultaneously, matching Figure 1A. As expected, SNR gets worse with finer in‐plane resolutions. To accommodate different body habitus, we opted for an in‐plane resolution of 2.2x2.2 mm^2^.

**Video S3:** Representative SB and SMS results synchronized over one heartbeat from a healthy volunteer, movie of Figure 2. The color blocks indicate the SMS slices acquired simultaneously, matching Figure 1A. The SMS images provide sufficient blood‐myocardium contrast and capture the underlying dynamics. At mid‐diastole, the SMS and SB image contrasts are comparable. At end‐systole, SMS exhibits lower blood‐myocardium contrast than SB. This reduction did not hinder the evaluation of wall motion or volumes.

**Video S4:** SMS results synchronized over one heartbeat from two subjects with different body habitus, movie of Figure 3. The color‐coded blocks represent the SMS slices acquired simultaneously, matching Figure 1A. The top row shows a healthy volunteer (S1, female, 21 years old, BMI 20, heart rate 85 bpm). The bottom row shows a patient (S2, male, 40 years old, BMI 34, heart rate 70 bpm) with a history of myocardial infarction and previous sternal wire sutures (red arrows). The LV wall motion abnormalities observed in volunteer S2 are shown with blue arrows. Both displays provide sufficient blood‐myocardium contrast to capture the underlying dynamics. For S2, the metal artifacts are contained in size due to lower susceptibility at mid‐field.^37^

**Video S5:** RT‐SMS images and intensity vs. time plots from a volunteer experiencing irregular rhythms for approximately seven seconds of acquisition, movie of Figure 4. The delayed beat is highlighted in the line‐intensity plots (blue overlay). Irregular event is captured in all three slices without manual synchronization, showing PVC in apical, mid, and basal short‐axis slices.

**Video S6:** An automatic segmentation of epicardial and endocardial borders in all SMS and SB slices for two volunteers, over a heartbeat. S1 and S2 correspond to Figure 3 showing volunteers with high and low ES scores. The segmentation was done via Medviso Segment for research.^38^ Note that the contrast in the low‐score SMS images was adequate for an automatic segmentation tool. The estimated volumes for S1: 82.57/83.94 mL at end‐diastolic volume (**EDV**) for SMS and SB, respectively, and 49.14/48.73 mL at end‐systolic volume (**ESV**) for SMS and SB, respectively. The estimated volumes for S2: 126.00/127.2 at EDV and 82.94/76.99 at ESV for SMS and SB, respectively.

**Video S7:** Reduction of “ripple‐like” artifacts in SMS RT‐images from a healthy volunteer using a spiral artifact reduction algorithm. In a few isolated cases, “ripple‐like” artifacts are present in the SMS images, as shown in the top row (red arrows). This is likely due to some remaining cross‐talk between the slices, and/or a region of high signal intensity (such as blood or fat) that is outside the primary field‐of‐view and produces an aliasing artifact. The latter artifact can be eliminated by employing a spiral aliasing reduction method.^39^ Here, the mid‐row is used as a manual input. Then, reconstruction was run again with the artifact reduction to produce the bottom row where the aliasing artifact is suppressed without a significant loss in the image quality or SNR.

**Figure S1:** Line intensity profiles of two subjects with different body habitus. The volunteers correspond to Figure 3 and Video S4. Note that the display windows have been chosen differently in SMS and SB images. This was done to compensate for the loss of contrast, as it can be misinterpreted for spatiotemporal smoothing.
**Figure S2:** SMS images at mid‐diastole and end‐systole from two subjects with different edge sharpness scores. The semi‐automated segmentation is overlayed in the end‐systole images of S2. Volunteer S1 shows higher contrast at mid‐diastole (17.59) and end‐systole (12.15). S2 scored worst at mid‐diastole (7.79) and end‐systole (7.65). In S2, the ES score is found low. This is because of the lower blood‐myocardium contrast but also because of the papillary muscles. In some cases, the ES method measures papillary muscle‐myocardium contrast instead of blood‐myocardium contrast (S10–S12). This contributes to the underestimation of the true sharpness.

## Data Availability

Sample reconstruction code and sample raw data with shorter acquisition time are available on GitHub: https://github.com/usc‐mrel/SMS‐RT. A snapshot of the reconstruction code is available via Zenodo: https://zenodo.org/records/12727826. Full sample raw data is available via Zenodo: https://zenodo.org/records/12737931.
