# Supplementary material for: Simultaneous multi‐slice cardiac real‐time MRI at 0.55T
Source: Magn Reson Med. 2024 Nov 6;93(4):1723–32. doi: 10.1002/mrm.30364 (PMC11782716; doi:10.1002/mrm.30364)
Supplement: Supplementary file 8 — Figure S1: Line intensity profiles of two subjects with different body habitus. The volunteers correspond to Figure 3 and Video S4. Note that the display windows have been chosen differently in SMS and SB images. This was done to compensate for the loss of contrast, as it can be misinterpreted for spatiotemporal smoothing. Figure S2: SMS images at mid‐diastole and end‐systole from two subjects with different edge sharpness scores. The semi‐automated segmentation is overlayed in the end‐systole images of S2. Volunteer S1 shows higher contrast at mid‐diastole (17.59) and end‐systole (12.15). S2 scored worst at mid‐diastole (7.79) and end‐systole (7.65). In S2, the ES score is found low. This is because of the lower blood‐myocardium contrast but also because of the papillary muscles. In some cases, the ES method measures papillary muscle‐myocardium contrast instead of blood‐myocardium contrast (S10–S12). This contributes to the underestimation of the true sharpness. [file MRM-93-1723-s003.docx]

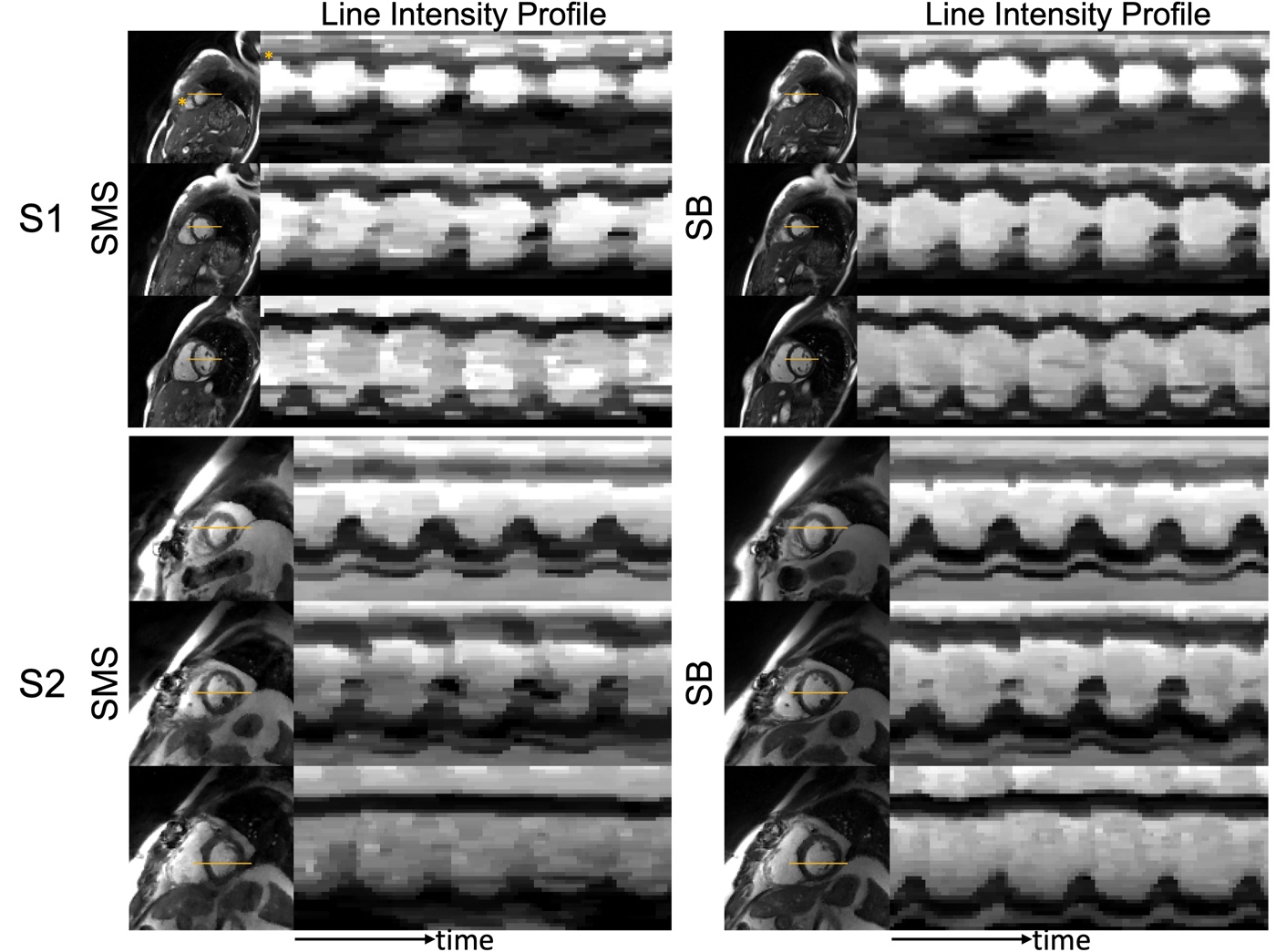


**Supporting Information Figure S1: Line intensity profiles of two subjects with different body habitus.** The volunteers correspond to **Figure 3** and **Supporting Information Video S4**. Note that the display windows have been chosen differently in SMS and SB images. This was done to compensate for the loss of contrast, as it can be misinterpreted for spatiotemporal smoothing.


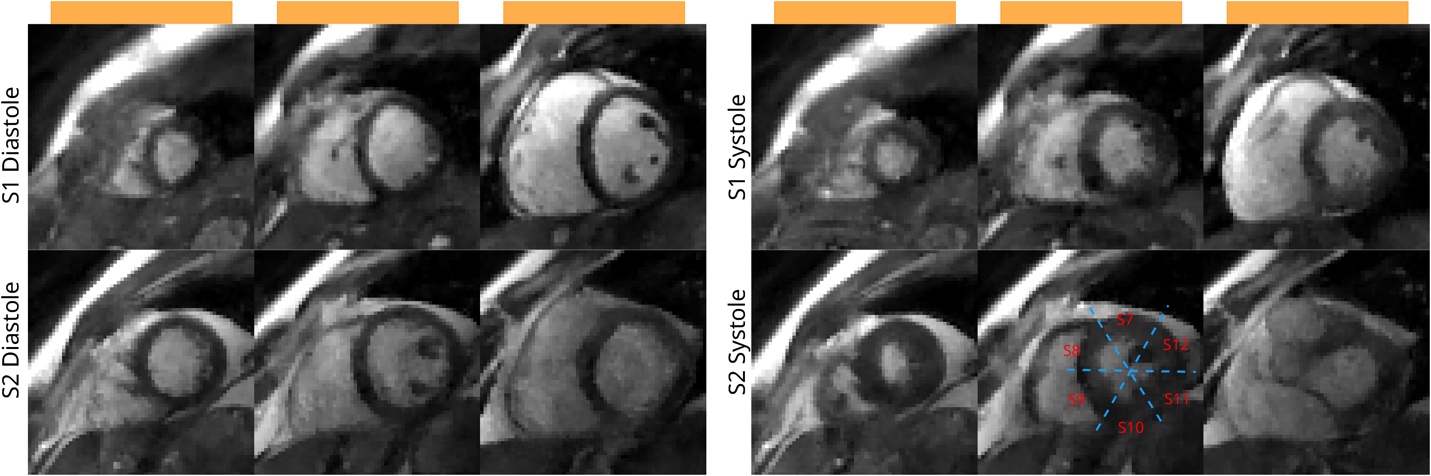


**Supporting Information Figure S2: SMS images at mid-diastole and end-systole from two subjects with different edge sharpness scores.** The semi-automated segmentation is overlayed in the end-systole images of S2. Volunteer S1 shows higher contrast at mid-diastole (17.59) and end-systole (12.15). S2 scored worst at mid-diastole (7.79) and end-systole (7.65). In S2, the ES score is found low. This is because of the lower blood-myocardium contrast but also because of the papillary muscles. In some cases, the ES method measures papillary muscle-myocardium contrast instead of blood-myocardium contrast (S10 – S12). This contributes to the underestimation of the true sharpness.
